# Delayed Loop Ileostomy Reversal Increases the Risk of Colitis and *Clostridium difficile* Infection

**DOI:** 10.1111/ans.70838

**Published:** 2026-07-01

**Authors:** Yan S. Tan, S. Fujino, Marcus E. H. Chan, Johann W. Z. Leh, Asiri M. K. Arachchi, V. Narasimhan, Thomas S. Suhardja, Hanumant S. Chouhan, Pathirajage C. K. Saranasuriya, James T. Lim, Thang C. Nguyen, William M. K. Teoh, Yeng K. Tay

**Affiliations:** ^1^ Department of Surgery (School of Clinical Sciences at Monash Health) Monash University Melbourne Australia

**Keywords:** *Clostridium difficile* infection, colitis, loop ileostomy, loop ileostomy reversal, obesity

## Abstract

**Background:**

Temporary loop ileostomy formation is a routine procedure performed to protect distal colorectal anastomosis. Traditionally, the ideal timing of ileostomy reversal is believed to fall between 3 and 6 months. It has been suggested that delayed reversal increases the risk of complications. Our study looks to demonstrate the relationship between delayed reversal and its associated risks in a real‐world setting.

**Methods:**

This is a retrospective cohort study conducted at Monash Health, analysing patients who underwent loop ileostomy reversal from May 2016 to December 2023. Main outcome measures include incidence of post‐reversal complications, including colitis, *Clostridium difficile* infection, anastomotic leak, ileus, wound infection and acute kidney injury. Secondary outcome is to investigate the impact of COVID‐19 on ileostomy reversal timing.

**Results:**

A total of 223 patients were included. The primary indications for initial surgery included cancer (*n* = 134), inflammatory bowel disease (*n* = 19) and diverticular disease (*n* = 29). The median time between initial surgery and ileostomy reversal was 341 days (IQR = 211–530). Post‐reversal complications occurred in 65 patients (29.1%). Patients reversed after the pandemic experienced a delay between surgeries (median: 411, IQR = 276–660) compared with those prior (median: 294, IQR = 196–408, *p* < 0.0001). A statistically significant association was seen between delayed reversal and post‐operative complications (OR 1.11, *p* = 0.020, ROC AUC = 0.588). Ileostomy reversal occurring beyond 134 days (based on the Youden's Index with 90% sensitivity‐based cutoff) is associated with increased risk of complications.

**Conclusion:**

Delayed loop ileostomy reversal is associated with post‐operative complications specifically colitis and *C. difficile* colitis.

## Graphical abstract

Delayed loop ileostomy reversal is associated with increased post‐operative complications specifically colitis and *Clostridium difficile* colitis. The COVID‐19 pandemic caused a significant delay in ileostomy reversal timing.

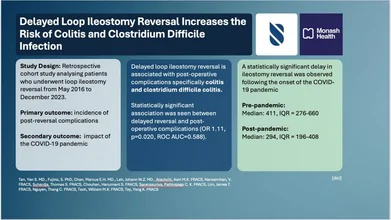

## 1 Introduction

Temporary loop Ileostomy formation is a well‐recognised routine procedure in colorectal surgery for defunctioning to protect distal colorectal anastomosis and reduce the risk and severity associated with anastomotic leak.

Stoma‐related morbidity is a notable complication post‐ileostomy formation with high incidences of parastomal hernia, acute kidney injury (AKI) and peristomal skin complications with major impact on quality of life. Its prevalence could be up to 10%–30%, further exacerbated by the COVID‐19 pandemic [1]. Evidence has shown that the longer a stoma remains in situ, the higher the risk of developing stoma‐related complications. Hence, it is important to balance anastomotic healing and preventing stoma‐related complications.

Traditionally, the ideal timing of ileostomy reversal is believed to fall between 3 and 6 months, although this remains debatable to date. It has been suggested that delayed ileostomy reversal increases the risk of post‐operative complication and hospital length of stay [2, 3, 4, 5, 6]. Furthermore, recent studies have proposed that early reversal (8–13 days after resection) is safe in selected patients [1, 7].

Due to the COVID‐19 pandemic, the Australian healthcare system struggled with significant delays in elective surgery waiting times, with an unprecedented doubling of waiting times observed between March 2020 and March 2022 [8]. The many restrictions to surgical services during that period led to an increasing backlog of elective surgeries. In our hospital system, elective ileostomy reversals are typically waitlisted as Category 2 (within 90 days) after preoperative workups assessing distal anastomotic healing. However, a significantly longer waiting time for ileostomy reversals is observed during the pandemic, imposing negative impacts on patient outcomes.

For this study, we conducted a retrospective cohort study in a single‐centre tertiary hospital in Melbourne, Australia. The relationship between ileostomy reversal timing and its impact was examined to ascertain if delayed reversal is associated with increased risks of complications. Furthermore, we have investigated the impact of COVID‐19 on ileostomy reversal timing in our centre.

## 2 Methods

### 2.1 Study Design and Patient Selection

This is a retrospective cohort study conducted at Monash Health, analysing patients who underwent loop ileostomy reversal from May 2016 to December 2023. Ethics approval was obtained from the local health network. The study period was selected to include both pre‐pandemic and pandemic phases to allow secondary analysis of the impact of COVID‐19 on reversal timing. Patients were included if they had a loop ileostomy formation following initial colorectal surgery and subsequently underwent reversal. Exclusion criteria included patients who did not undergo reversal, patients who received an end ileostomy or had missing key clinical data. Patient characteristics, surgical details and post‐operative outcomes were collected from electronic medical records.

### 2.2 Variables and Definitions

The primary outcome was the incidence of post‐reversal complications, including colitis, *Clostridium difficile* (CD) colitis, anastomotic leak, ileus, wound infection and AKI. Colitis was defined based on clinical symptoms (e.g., diarrhoea and abdominal pain) in conjunction with radiological or endoscopic findings. CD colitis was defined by a positive stool test for CD infection. Anastomotic leak was defined by radiological, clinical or intraoperative evidence of leakage at the ileostomy reversal anastomosis. Ileus was diagnosed based on clinical and imaging findings. Wound infection was defined as surgical site infection requiring intervention (e.g., antibiotics, wound management or return to theatre). AKI was defined according to the Kidney Disease: Improving Global Outcomes (KDIGO) criteria [9]. The primary exposure variable was the time between initial surgery and ileostomy reversal. Additional variables included age, sex, BMI, reason for initial surgery, surgical urgency (emergency vs. elective) and readmission after initial surgery. To assess the impact of the COVID‐19 pandemic, patients were categorised into pre‐pandemic (before February 2020) and post‐pandemic (after March 2020) groups based on the Australian lockdown timeline.

### 2.3 Comparison of Groups

Continuous variables were reported as median with interquartile range (IQR) due to the non‐normal distribution of key variables, including time to reversal. Comparisons between groups were performed using the Wilcoxon rank‐sum test. Categorical variables were compared using the chi‐squared test or Fisher's exact test, as appropriate depending on cell counts.

### 2.4 Association Between Ileostomy Reversal Timing and Complications

Logistic regression analysis was performed to evaluate the association between time to ileostomy reversal and post‐reversal complications. Time to reversal was analysed as a continuous variable. Odds ratios (ORs) with 95% confidence intervals (CI) were calculated and, for clinical interpretability, expressed per 100‐day increase. Receiver operating characteristic (ROC) curve analysis was conducted as an exploratory analysis to assess the relationship between reversal timing and complications. Cut‐off values were estimated using the Youden index and a sensitivity‐based approach (90% sensitivity) to reflect different clinical priorities.

### 2.5 Subgroup Analysis

Subgroup analyses were performed for colitis and CD colitis to further evaluate associated factors. The impact of the COVID‐19 pandemic on reversal timing was assessed using the Wilcoxon rank‐sum test.

A *p*‐value < 0.05 was considered statistically significant. All statistical analyses were performed using JMP Pro 17 (SAS Institute Inc., Cary, NC, USA).

## 3 Results

### 3.1 Patient Characteristics

A total of 223 patients were analysed, consisting of 134 males and 89 females. The median age was 60 years (range: 17–84), and the median BMI was 26.4 (range: 16.4–47.8). The primary indications for initial surgery included cancer (*n* = 134), inflammatory bowel disease (IBD) (*n* = 19) and diverticular disease (*n* = 29), amongst others. Emergency surgery was performed on 73 patients, whilst 150 underwent elective procedures. Readmission after initial surgery was recorded in 82 patients (36.8%). The median time between initial surgery and ileostomy reversal was 341 days (IQR = 211–530), and post‐reversal complications occurred in 65 patients (29.1%) (Table 1).

**TABLE 1:** Patients background.

Factor	Value
Sex (male/female)	134/89
Age	60 (17–84)
BMI	26.4 (16.4–47.8)
Reason for initial surgery	Cancer: 134, IBD: 19, diverticular: 29, intestinal perforation: 16, intestinal ischaemia: 2, perianal infection: 4, anastomotic leakage: 4, fistula: 3 and others: 12
Initial surgery	Emergency: 73
	Elective: 150
Readmission after initial surgery	Yes: 82
	No: 141
Median days between initial surgery and reversal	341 (range: 12–2646)
Post‐reversal complications	Yes: 65
	No: 158

### 3.2 Impact of COVID‐19 Pandemic on Ileostomy Reversal Timing

A significant delay in ileostomy reversal was observed following the onset of the COVID‐19 pandemic. The shutdown in Australia began in March 2020, thus the analysis was conducted by comparing patients who underwent reversal before the pandemic (before February 2020) and after the pandemic (March 2020 onwards). Patients who had their ileostomy reversal after the pandemic experienced a significantly longer interval between initial surgery and reversal (median: 411, IQR = 276–660) compared with those before the pandemic (median: 294, IQR = 196–408, *p* < 0.0001).

### 3.3 Association Between Ileostomy Reversal Timing and Complications

Logistic regression analysis demonstrated a significant association between delayed ileostomy reversal and post‐reversal complications (OR 1.11, *p* = 0.020, ROC AUC = 0.588). Amongst specific complications, colitis (OR 1.26, *p* = 0.001, ROC AUC = 0.711) and CD colitis (OR 1.20, *p* = 0.011, ROC AUC = 0.785) demonstrated strong associations with prolonged ileostomy duration (Table 2).

**TABLE 2:** Association between delayed ileostomy reversal and post‐reversal complications.

Outcome	OR (per 100‐day increase)	95% CI	*p*	ROC AUC	Youden cutoff (days)	Sensitivity cutoff (days)
All complications	1.11	1.02–1.23	0.020	0.588	268	134
Colitis	1.26	1.10–1.46	0.001	0.711	532	269
CD colitis	1.20	1.03–1.39	0.011	0.785	414	133

### 3.4 Identifying Ileostomy Reversal Timing Threshold

To minimise post‐reversal complications, both Youden's Index and sensitivity‐based cutoffs were evaluated. Youden's Index determined the optimal cutoff for reversal to be 268 days for all post‐reversal complications, 532 days for colitis and 414 days for CD colitis. However, upon prioritising a 90% sensitivity threshold, earlier reversal is recommended: 134 days for all complications, 269 days for colitis and 133 days for CD colitis (Table 1).

### 3.5 Association Between Clinical Factors and Complications

Post‐reversal complications were significantly associated with patient age, with those experiencing complications being older (*p* = 0.020) (Table 3). Specifically, there was a significant association between incidences of CD colitis and BMI (*p* = 0.023) (Table 3). However, no significant differences were observed for sex, surgical indication and emergency surgery. Patients who developed colitis (*n* = 13) had a significantly longer duration between initial surgery and ileostomy reversal compared with those without colitis (560 vs. 336 days, *p* = 0.011) (Table 4). Similarly, patients with CD colitis (*n* = 7) had significantly higher BMI (*p* = 0.023) and longer ileostomy duration (578 vs. 337 days, *p* = 0.010), though no other clinical factors were significantly associated (Table 5).

**TABLE 3:** Association between complications and clinical factors.

Factors	Post‐reversal complications (*n* = 65)	No post‐reversal complications (*n* = 158)	*p*
Sex (male/female)	38/27	96/62	0.750
Age	65 (55–72)	58 (48–68)	0.020
BMI	26.1 (22.3–30.6)	26.5 (22.6–30.2)	0.801
Reason for initial surgery (cancer/not)	39/26	95/63	0.986
Reason for initial surgery (IBD/not)	2/63	17/141	0.080
Reason for initial surgery (diverticular/not)	12/53	18/140	0.160
Initial surgery (emergency/elective)	24/41	49/109	0.393
Readmission after initial surgery (yes/no)	30/35	52/106	0.062
Days between initial surgery and reversal	393 (283–578)	322 (202–469)	0.039

**TABLE 4:** Association between colitis and clinical factors.

Factors	Colitis (*n* = 13)	Non‐colitis (*n* = 210)	*p*
Sex (male/female)	7/6	127/83	0.782
Age	63 (45–76)	60 (50–69)	0.478
BMI	26.1 (23.5–34.1)	26.4 (22.5–30.3)	0.462
Reason for initial surgery (cancer/not)	7/6	127/83	0.782
Reason for initial surgery (IBD/not)	1/12	18/192	0.912
Reason for initial surgery (diverticular/not)	1/12	29/181	1.000
Initial surgery (emergency/elective)	4/9	69/141	0.777
Readmission after initial surgery (yes/no)	8/5	74/136	0.058
Days between initial surgery and reversal	560 (346–945)	336 (207–486)	0.011

**TABLE 5:** Association between CD colitis and clinical factors.

Factors	CD colitis (*n* = 7)	Non‐CD (*n* = 216)	*p*
Sex (male/female)	3/4	131/85	0.706
Age	63 (48–79)	60 (50–69)	0.469
BMI	33.8 (25.5–37.0)	26.3 (22.4–30.1)	0.023
Reason for initial surgery (cancer/not)	5/2	129/87	0.534
Reason for initial surgery (IBD/not)	0/7	19/197	0.412
Reason for initial surgery (diverticular/not)	1/6	29/187	0.948
Initial surgery (emergency/elective)	1/6	72/144	0.291
Readmission after initial surgery (yes/no)	4/3	78/138	0.419
Days between initial surgery and reversal	578 (414–933)	337 (208–492)	0.010

## 4 Discussion

Our study has shown that delayed ileostomy reversal is associated with increased risk of complications, which is consistent with the current literature [1, 2, 3, 4, 5]. Our study has demonstrated a statistically significant association between delayed reversal with colitis and CD colitis.

### 4.1 CD Colitis

Previous studies have proven the association between CD colitis and post‐loop ileostomy reversals which is consistent with our current study [10, 11, 12]. A retrospective cohort study demonstrated a 5.6% incidence of CD colitis post‐ileostomy closure with multivariable analysis portraying a nearly sevenfold increase in odds of CD colitis with delayed reversal (> 365 days) [11]. The pathophysiology behind this is unknown. However, it is believed that it could be related to CD colonisation in the disused colon post‐formation of ileostomy, proven with several studies demonstrating high CD colitis incidences in ileostomy reversal compared with other colorectal procedures [10, 11, 12].

Our subgroup analysis has demonstrated a significant relation between increased BMI and incidence of post‐reversal CD colitis. Current literature has shown mixed outcomes between the significance of an increased BMI and incidence of CD colitis post reversal [10]. A systematic review in 2022 has revealed a negative association between BMI and CD colitis; however, a case–control study in 2013 has demonstrated otherwise [13, 14]. Despite this, a potential pathophysiological hypothesis has been raised. It is well known that patients with higher BMI are prone to developing sarcopenic obesity, where there is a loss of muscle mass and function associated with increased adiposity [15]. These individuals have higher adiposity which poses them to chronically increased systemic inflammatory response due to increased proinflammatory cytokine levels, increased leptin and reduced adiponectin levels [15, 16]. These factors in combination dampen the body's immune response towards infections.

### 4.2 Gut Microbiome Theory

Contributing factors towards post‐reversal colitis may include gut microbiome alterations and impaired intestinal renewal due to faecal diversion. Animal studies have shown a substantial distortion of intestinal muscular and mucosal architecture in the distal colon following faecal stream diversion, which is believed to be due to cellular nutrient deprivation and lack of luminal stimulation [17, 18]. This nutritional deprivation has been hypothesised to contribute to nutritional deprivation in gut microbes [17]. Intestinal dysbiosis has also been linked to a proinflammatory state within the intestine which predisposes it to infection [17]. A systematic review in 2021 has shown that faecal diversion results in a loss in gut microbiome diversity which increases the risk of colitis, further reinforcing the role of gut microbiome alteration in the pathophysiology of post‐reversal colitis [19].

### 4.3 Safe Reversal Timing

Whilst there is no universally agreed optimal timing for safe ileostomy reversal, the consensus was that reversal can be conducted safely between 3 and 6 months post‐initial surgery. According to our data, reversal happening beyond 268 days based on the Youden's Index cutoff, and 134 days by using sensitivity‐based cutoff (90%) is associated with higher risk of complications. These values prioritise prevention of complications by capturing high‐risk patients earlier. This is consistent with the general recommendation in literature. It suggests that earlier reversal may be vital in reducing overall morbidity. Nonetheless, patient factors should be considered when planning for early stoma reversal, including the patient's overall health, need for additional treatment (e.g., chemoradiotherapy) or presence of complications post‐initial surgery. Therefore, individualised decision‐making should be conducted with patients prior to reversal.

### 4.4 Impact of COVID‐19

During the COVID‐19 pandemic, Melbourne became the longest locked‐down city in the world with a significant compromise to the elective surgery waitlist [8]. Our study has demonstrated that the ileostomy reversal timing in our centre was significantly delayed during the COVID‐19 pandemic (411 vs. 294 days, *p* < 0.0001). As ileostomy reversal is mainly conducted in an elective setting, its delays during the pandemic are a true reflection on the impacts of the pandemic on elective surgery. The Victorian state response during the pandemic involved strict social and contact restrictions, combined with the financial and workforce strain on the system. This meant elective surgery was put on hold for periods of time, causing a significant backlog of cases. Following the pandemic, the state has announced a surgical recovery and reform programme that has allowed funding to deliver additional elective procedures per year in response [8]. The pandemic highlights the gap in the Australian healthcare system in adapting to healthcare catastrophes. The system needs to place emphasis on streamlining a programme that allows a smooth delivery of elective surgery [8]. This is to ensure patient safety and optimal delivery of evidence‐based care. As our data has shown that delayed reversal has a negative impact towards patient outcomes, this urges the healthcare system to explore strategies to ensure reversals happen within the optimal timing to minimise post‐operative morbidity.

### 4.5 Potential Clinical Implications

Pre‐reversal interventions have been an emerging topic nowadays, with ongoing research looking into the role of therapies targeting different aspects of the believed pathophysiology contributing to post‐ileostomy reversal complications.

A previous randomised controlled trial in 2017 and the current ILEOSTIM trial look into mechanical stimulation of the efferent loop stimulation prior to reversal surgery, as a method to improve efferent limb musculo‐vili atrophy [20, 21]. Another randomised controlled trial in Brazil is evaluating the role of rectal stimulation with prebiotics and probiotics to investigate its role in post‐reversal complications [22]. It would perhaps provide further insight once the findings of both studies are published.

A few studies have investigated the role of probiotics in pre‐ileostomy reversal optimization. Interestingly, studies that recruited patients with cancer as an indication for diversion ileostomy had no significant differences with probiotics pre‐operatively [23, 24]. However, a study that looked into ileostomy closure in patients with previous proctocolectomy and ileal pouch formation demonstrated a significant decrease in post‐ileostomy reversal incidence of pouchitis in patients that received oral probiotics [25]. This suggests that there is a promising role of probiotics in specific patient populations.

Despite the lack of current clinical evidence, another promising intervention is the role of short chain fatty acid (SCFA) enemas for its potential benefits on treatment of colitis [26, 27, 28]. Multiple studies have shown that diversion colitis is strongly associated with the lack of SCFA in the diverted colon, potentially due to the altered microbiome [29, 30]. We know that SCFAs such as butyrate enhance intestinal barrier function and mucosal immunity through their role in colonocyte metabolism, which highlights the potential role of SCFA in reducing the incidence of colitis post‐ileostomy reversal [26, 29]. However, better quality studies would have to be conducted.

### 4.6 Limitations

This study was conducted in a single‐centre setting, where our findings may not apply to other institutions with different surgical practices and patient populations. The retrospective study design allows for potential selection bias and residual confounding factors as data was collected through past medical records. Several variables possibly contributing towards post‐operative complications such as nutritional status, steroid use, background comorbidities and more were not considered. Additionally, factors leading to delayed reversal should also be considered. These include adjuvant chemoradiotherapy, which significantly impacts gut microbiome, the need for anastomotic dilation and other medical reasons (such as delayed recovery or complications from initial surgery, unexpected readmission to hospital) [29].

Moreover, the median timing of reversal in our patient cohort is 341 days, which is well over the current recommended 3–6 months due to the limitations of a public system and COVID‐19 delays. Hence, it can be thought that most patients are undergoing late ileostomy reversals, limiting the comparison for early versus late reversals given the limited number of patients having reversals < 6 months. Furthermore, the small sample size in the complication subgroups would limit statistical power. Given the variable data and conclusions in the literature, these findings should be interpreted with care. Ideally, further exploration into this specific outcome with well‐designed randomised controlled trials should be conducted.

Our dataset has a heavy overlap with the COVID‐19 pandemic timeline, thus the factor of pandemic‐related variability in terms of elective surgery timing and delays. This disruption to healthcare access may limit generalizability in future non‐pandemic settings; hence consideration should be taken when interpreting results.

In conclusion, delayed loop ileostomy reversal is associated with post‐operative complications specifically colitis and CD colitis. The COVID‐19 pandemic caused a significant delay in ileostomy reversal timing. This reflects the gap in the Australian public healthcare setting to adapting to healthcare catastrophes, particularly in an elective surgery setting.

## Author Contributions

**S. Fujino:** data curation, conceptualization, investigation, writing – original draft, methodology, validation, visualization, writing – review and editing, formal analysis, project administration, supervision, resources. **Yan S. Tan:** conceptualization, writing – original draft, methodology, visualization, writing – review and editing, formal analysis, project administration, validation, data curation, resources. **Hanumant S. Chouhan:** conceptualization, validation, formal analysis, supervision, writing – review and editing. **Thomas S. Suhardja:** conceptualization, validation, formal analysis, supervision, writing – review and editing. **Johann W. Z. Leh:** conceptualization, writing – original draft, writing – review and editing, data curation, validation. **V. Narasimhan:** conceptualization, validation, formal analysis, supervision, writing – review and editing. **Yeng K. Tay:** conceptualization, writing – review and editing, supervision, validation, formal analysis. **William M. K. Teoh:** conceptualization, validation, writing – review and editing, formal analysis, supervision. **Marcus E. H. Chan:** conceptualization, writing – original draft, writing – review and editing, data curation, validation. **Asiri M. K. Arachchi:** conceptualization, validation, formal analysis, supervision, writing – review and editing. **Pathirajage C. K. Saranasuriya:** conceptualization, validation, formal analysis, supervision, writing – review and editing. **James T. Lim:** conceptualization, validation, formal analysis, supervision, writing – review and editing. **Thang C. Nguyen:** conceptualization, validation, formal analysis, supervision, writing – review and editing.

## Conflicts of Interest

The authors declare no conflicts of interest.

## Data Availability

The data that support the findings of this study are available from the corresponding author upon reasonable request.
